# Decreasing the number of arthroscopies in knee osteoarthritis – a service evaluation of a de-implementation strategy

**DOI:** 10.1186/s12891-020-3125-8

**Published:** 2020-03-03

**Authors:** Timothy Barlow, Timothy Rhodes-Jones, Sue Ballinger, Andrew Metcalfe, David Wright, Peter Thompson

**Affiliations:** 1 University Hospitals of Coventry and Warwickshire, Clifford Bridge Road, Coventry, CV2 2DX UK; 2 Clinical Sciences Research Laboratories, Warwick University UHCW, Clifford Bridge Road, Coventry, CV2 2DX UK

**Keywords:** Knee osteoarthritis, Conservative care, Arthroscopy

## Abstract

**Background:**

The Personalised Knee Improvement Programme (P-KIP) was developed based on previously published work, with the hypothesis that surgeons would refer patients to a well-structured conservative management intervention instead of for arthroscopy (de-implementation of arthroscopy by substitution with P-KIP). This meets NICE guidelines and international recommendations but such programmes are not widely used in the UK. Our aim was to determine whether P-KIP would reduce the number of arthroscopies performed for knee osteoarthritis.

**Methods:**

P-KIP is a conservative care pathway including a group education session followed by individually tailored one-to-one dietician and physiotherapy sessions. Virtual clinic follow-up is conducted three to 6 months after completion of the programme. The service began in July 2015.

The number of arthroscopies saved, measured from hospital level coding data, is the primary outcome measure. Interrupted time series analysis of coding data was conducted. As a quality assurance process, patient reported outcome measures (Oxford Knee Score; Euroqol 5D) were collected at baseline and at follow up.

**Results:**

Time series analysis demonstrates that the programme saved 15.4 arthroscopies a month (95% confidence interval 9–21; *p* < 0.001), equating to 184 arthroscopies a year in a single hospital. The PROMs data demonstrated improvements in patient reported outcome scores consistent with previous published reports of conservative interventions in similar patient populations.

**Conclusions:**

Results suggest that P-KIP reduces the number of arthroscopies performed, and patients who took part in P-KIP had an improvement in their knee and general health outcomes. P-KIP has the potential to deliver efficiency savings and relive pressure on operative lists, however replication in other sites is required.

## Background

Knee osteoarthritis (OA) is a common condition, affecting more than 10% of the population over 60 years old [1]. A traditional treatment option for knee OA has been knee arthroscopy. However Moseley et al. conducted a much publicised sham randomised controlled trail demonstrating no benefit of arthroscopy, and this, combined with a Cochrane review of the literature up to 2006, resulted in NICE recommending that arthroscopy should not be used in knee osteoarthritis [2–4]. This guidance was updated in 2014 [5], with multiple randomised controlled trials, systematic reviews, and meta-analyses demonstrating consistent results [6, 7],

Other bodies have released guidance on arthroscopy in knee osteoarthritis with similar recommendations [4, 8, 9]. However, the data from such papers still suggests a significant number of arthroscopies are being performed for osteoarthritis [10–14].

In collaboration with NICE, we investigated barriers to the implementation of the guidance from both orthopaedic surgeons and patients [15]. This work was based in the Theoretical Domains Framework, which allowed for the development of strategies targeted to the specific barriers [15]. The barriers identified included a:

- Perceived pressure from patients to do something (with arthroscopy perceived as the management that was desired).
- Patients’ desire for something to be done, but with generally no fixed view on what that “something” should entail (although “more physiotherapy” was not desirable for a proportion of people).
- Limited other options available. A widely-held view from patients was that they had “already tried physiotherapy”. This made current referral pathways problematic, as there were limited other options available, and surgeons were then under pressure (perceived or otherwise) to offer something else.
- A desire to meet patients’ expectations
- Perceived time pressure upon surgeons in busy clinics

The Personalised Knee Improvement Programme (P-KIP) was designed to address these barriers. The referral process was an online referral embedded in established clinical systems that took less than 1 minute to complete, and was accompanied by high quality, professionally printed pamphlets and patient atlases (to facilitate the feeling that this was not something that patients had been through before). There is some evidence that such targeted interventions have greater effects [16].

Additionally, P-KIP could be used as an alternative (or a substitute) for arthroscopy. A specific feeling among surgeons was a lack of alternative treatments, along with patients wanting “something” done (although not just “more physiotherapy”). Therefore, we designed P-KIP to offer a substitute pathway that surgeons and patients felt was an acceptable alternative. A growing body of evidence on de-implementation strategies (reducing low value care) has emerged in recent years [17–19], with one strategy (based on psychological models of cognition) suggesting that substitution may facilitate the de-implementation of low value care [20].

Therfore, P-KIP’s aim was to offer an accessible, evidence based conservative care pathway as an alternative to knee arthroscopy, and was developed in collaboration with physiotherapists, dietitians, orthopaedic surgeons, and members of the public. Our hope was that such a programme would improve patient care, relieve pressure on theatre capacity [21], and deliver efficiency savings in line with NHS targets [22].

The aim of this paper is to investigate the effect the programme had on the number of arthroscopies performed.

## Methods

### The P-KIP pathway

Patients with knee osteoarthritis seen in orthopaedic clinics can be referred to P-KIP. Such patients have been referred on to secondary care within a university teaching hospital within the UK. Patients with knee osteoarthritis that are considered by the surgeon to be candidates for knee arthroscopy using traditional criteria can be referred to the service by the orthopaedic surgeon. The eligibility criteria are judged by the referring surgeon in a pragmatic manor:

#### Inclusion

- Over 45 years
- Confirmed diagnosis of osteoarthritis
- Historically patient would have been a candidate for Arthroscopy
- Able to engage in targeted physiotherapy and dietary change

#### Exclusion

- Significant hip or back pathology
- Previous arthroscopy (last 2 years) on affected knee

P-KIP teaches self-management of knee osteoarthritis and is delivered by a multidisciplinary team of physiotherapy technicians, physiotherapists, dieticians, and orthopaedic surgeons within the secondary care setting. Patients flow through in a stepwise manner:

1. A group session providing information on core interventions and directly challenging the misconceptions of osteoarthritis being a “wear and tear” disease that is “doomed to get worse”. Instead patients are taught the “wear, tear, repair” model, popularised by Arthritis Research U.K. [23] Detailed information is provided with a high quality patient atlas, providing a record for all steps of the patients’ journey. Baseline patient reported outcome measures are also collected.

2. Individual sessions with both dietician and physiotherapist provide intensive therapy with the aim of empowering patients to engage with behaviour changes. Both the exercise and dietary interventions have been designed specifically for patients with knee OA, and visits are co-ordinated to decrease the number of trips for the patients, and to allow discussion between physiotherapists and dietitian. The number and exact content of sessions is determined between the patient and the physiotherapist/dietitian, and altered to suit the individual. At the last appointment with either physiotherapist or dietitian patient reported outcome measures are collected.

3. Virtual clinic follow-up (telephone consultation) by peri-operative specialist practitioners (PSPs) completes the pathway. This follow up takes place between four and 6 months after the last face-to-face appointment. This appointment serves as an intervention to improve long-term adherence (although the evidence for this is questionable) [24], and allows open-ended patient feedback on the programme. All patients are discharged after P-KIP; however this consultation also acts as a “safety net”, with PSPs able to refer back to consultant clinic deemed appropriate at their clinical discretion.

Feedback of the overall results of P-KIP are provided to referring consultants, however individual results are available on request. P-KIP costs approximately £90,000 a year to run, and has a capacity of approximately 300 patients a year.

### Outcome measures

The primary outcome measure is the number of arthroscopies performed in the trust before and after P-KIP was introduced. This was measured from hospital coding data. Patients that had a knee arthroscopy performed for knee osteoarthritis between January 2014 to December 2017 were identified by interrogating the trust coding database. All patients over 45 that had knee arthroscopy were identified, but those patients that required a knee arthroscopy as described in the NICE guidance were excluded (i.e. locked knee, septic arthritis) – this accounted for less than 5% of the total number. The codes that were used to identify patients are described in Table 1 below. Eligibility and accuracy of coding data was then checked by the lead author for each case by screening the primary diagnosis code and the title of the operation.

**Table 1:** Codes used to identify knee arthroscopy procedures

Code	
W85.1 Endoscopic removal of loose body from knee joint	
W85.2 Endoscopic irrigation of knee joint	
W80.- Debridement and irrigation of joint plus	
Y76.7 Arthroscopic approach to joint plus	
Z84.6 Knee joint	
W85.8 Other specified therapeutic endoscopic operations on cavity of knee joint	
W83.3 Endoscopic shaving of articular cartilage plus	
Z84.6 Knee joint	
W82.2 Endoscopic resection of semilunar cartilage NEC	

### Quality assurance and cost effectiveness

There is a wealth of evidence on the effect of high-quality conservative care on this population of patients [2–7]. Therefore, as a quality assurance measure, we measured Patient Reported Outcome Measures at baseline and at their last face-to-face appointment. The measures used were the Oxford Knee Score and the EuroQuol 5 Dimension [25–27]. All patients with complete data that had been referred to P-KIP between the start date (July 2015) and December 2017 were included. As patients typically take a year to complete the programme from referral the last year of data is not reported.

Cost effectiveness was estimated using the savings generated by a decrease in the number of arthroscopies (using the national tariff system), offset against the cost of the programme.

### Statistical analysis

Normally distributed data is described with means and standard deviations, with non-parametric data displayed with medians and inter-quartile ranges. Normality was assessed informally using histograms.

A simple comparison of the rate of arthroscopy before and after P-KIP was not conducted. Solely comparing the means before and after P-KIP, without taking into account any trends within the data, may result in over or under estimating P-KIP’s effect [28]. Therefore interrupted time series analysis of coding data was conducted to assess the direct effect of P-KIP at 1 month and 6 months after implementation [28, 29]. All analyses were performed in SPSS version 22 [30].

**Fig. 1:**
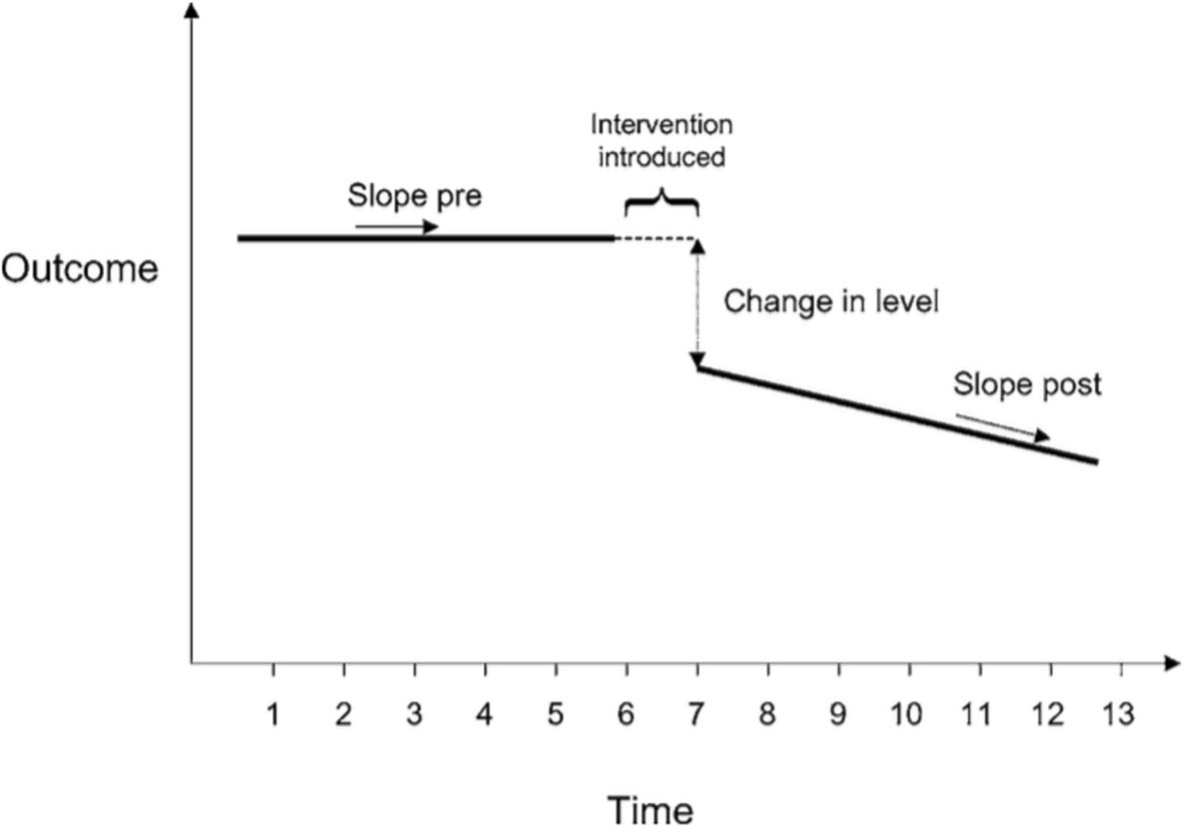
Change in slope and level investigated by a time series analysis. Figure reproduced with permission from Ramsey et al. (Cambridge University Press) [31]

A representation of a time-series regression analysis is displayed in Fig. 1 [31].

The analysis takes account of time trends and autocorrelation. As can be seen from Fig. 1, two effect sizes are acquired: the change in trend (slope of the line) before and after P-KIP; and the change in level (absolute change). According to Ramsay et al. [31], a change in level is defined as the difference between the observed level at the first intervention time point and that predicted by the pre-intervention time trend, and a change in trend is defined as the difference between post- and pre-intervention slopes. A negative change in level and slope would indicate a reduction in arthroscopy rates. The introduction of an intervention can take several months (and therefore the change in level will be different if measured at different time points).

Time series modelling was carried out in SPSS using the non-seasonal autoregressive expert modeller. The best fit pre and post-P-KIP lines were estimated using linear regression, and autocorrelation was adjusted for by using the maximum likelihood methods, with first-order autocorrelation tested for using the Ljung-box statistic. We first compared the slope change pre and post- P-KIP, and secondly we estimated the level change. To allow an estimate of the effect of the “bedding in” period on arthroscopy rates we performed two step change analysis: the 1 month effect which took account of all data from the time of implementation on; and the six-month effect, which excludes data for 6 months after the implementation (the “bedding in” period we expected). This was performed by extrapolating the pre-implementation regression line to the post-implementation regression line. The difference between these points gave a point estimate for the change in level. Full details of the model building procedure are available through the Cochrane Effective practice and Organisation of Care (EPOC) resource [32].

Comparison of outcome scores was conducted with the Wilcox Singed Rank Test for non-parametric data. No correction for multiple tests was conducted. Additionally, we have also performed paired T-test under the normality assumption on the same data [33]. All analysis was performed in SPSS version 22 [30].

## Results

Between July 2015 and December 2017, P-KIP had received 600 referrals. As it typically takes a year from referral to completion of programme, 377 participants had completed the programme at the time of analysis. Table 2 demonstrated the baseline demographics. Patients who completed the programme had on average 5 physiotherapy sessions and 2 dietician sessions.

**Table 2:** Baseline characteristics (*n* = 377 participants)

Age	61 (rage 45 to 81)
Female Gender	67%
Body Mass Index	33.3 (sd 6.8)
Oxford Knee Score	26 (IQR 14)
EQ-5D	70 (IQR 30)

### Number of arthroscopies

**Fig. 2:**
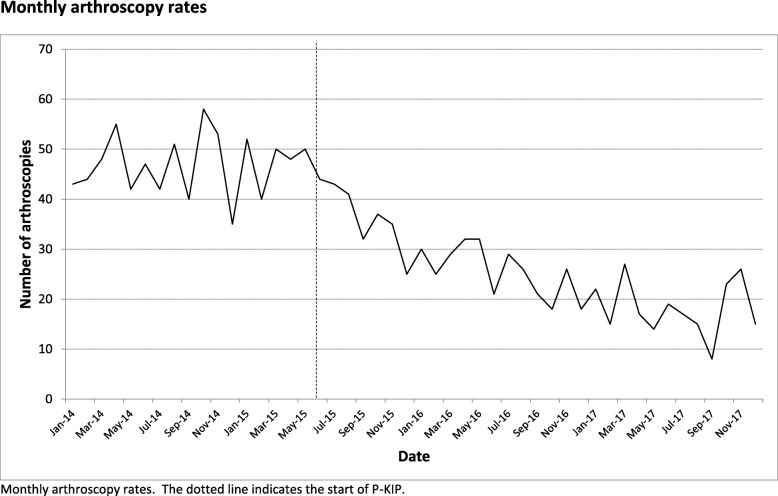
The number of arthroscopies performed by month. Dotted line represents the time of the intervention

Local hospital coding data was examined to determine the change in the number of arthroscopies done for patients with OA knee against NICE guidance. Figure 2 displays the number of arthroscopies from January 2014, 17 months before the programme began, to December 2017, 28 months after the programme began. Age and gender details for patients receiving arthroscopy before and after the introduction of P-KIP are displayed in Table 3 below.

**Table 3:** Baseline characteristics of patients receiving arthroscopy before and after P-KIP

	Age (mean)	Female Gender (%)
Before P-KIP (*n* = 466)	55	46
After P-KIP (*n* = 411)	53	44

Interrupted time series analysis was performed on the data from January 2014 to October 2016. The model (ARIMA (1,0,0); stationary R^2^ = 0.8; Ljung-box Q statistic = 17.2, *p* = 0.44) demonstrated the baseline (before P-KIP) arthroscopy rate was stable (coefficient of slope = 0.072; *p* = 0.688; 95% CI = − 0.284 - 0.428). The slope after P-KIP changes significantly (coefficient = −1.231; *p* < 0.001; 95% CI = − 1.779 to − 0.647), indicating the number of arthroscopies is decreasing significantly after P-KIP.

To further investigate this effect, the level change was determined at the 1 month (i.e. the effect including all data after P-KIP started) and 6 month intervals (i.e. not including the first 6 months after P-KIP started to remove the “bedding in” period). The analysis revealed that the number of arthroscopies decreased by 9.2 (95% CI = 3.7–14.7; *p* < 0.001) each month after P-KIP. Six-month effects (removing the “bedding in period”) revealed a decrease in arthroscopy rate of 15.4 (95% CI = 9.4–21.4; p < 0.001) per month: this equates to 184 arthroscopies a year (95% CI ranges between 108 and 252). The change in estimates suggests that, as expected, there was a bedding in period during P-KIP’s introduction.

P-KIP costs approximately £90,000 a year to run. With the current tariff for arthroscopies set at £1800 to £2700 (depending on co-morbidities) [34] results in an overall efficiency saving of approximately £300,000 (£240,000 to £400,000).

### Quality assessment

A flow chart of patients passing through the programme, Oxford Knee Scores (OKD), and EuroQuol 5 Dimension (EQ-5D) scores are available in the supplementary material. Outcome scores (both knee specific and general health) are comparable to previous published reports of high quality conservative care [2–7]. However, a dropout rate approximately 20% was encountered. Previous reports have rates of adherence with physiotherapy varying from 14 to 70% [35].

## Discussion

We describe the results from a conservative care pathway that was designed to decrease the number of arthroscopies performed for knee OA. This programme has successfully decreased the number of arthroscopies performed, with time series analysis suggesting it saves approximately 180 arthroscopies a year. Simultaneously, significant improvements in OKS and EQ-5D index scores were realised which is consistent with previous literature and suggests our P-KIP results in similar health outcomes [2–7]. We are not aware of any other programme specifically designed to decrease the number of arthroscopies for knee osteoarthritis, nor of any improvement project that has displayed this level of success.

This study is prone to various weaknesses. Our primary outcome measure was the number of arthroscopies – this number can theoretically alter dependent on the number and case mix of patients seen. Although arthroscopy rate (i.e. the number of arthroscopies performed as a percentage of new patients) would be preferable, the number of new patient referrals with condition specific coding is not captured routinely, and it is challenging to identify the number of patients who would potentially be eligible for P-KIP from this (i.e. some patients are discharged, some are offered total knee replacement). However, with increasing rates of knee osteoarthritis [1], this is likely to represent more patients with knee osteoarthritis being seen, although is it not possible to quantify this within our current system.

Our interrupted time series analysis is based on coding data. Coding data has been demonstrated to be inaccurate, with error rates between 1.1 and 45.8% reported, with an average of around 7% [36]. As a safeguard we went through data by hand, to ensure we were getting as accurate a picture as possible. Also, any error rate is likely to occur both pre- and post-P-KIP, and is therefore unlikely to affect the results significantly.

Further weaknesses include the use of longitudinal data, which is prone to secular trends. There was also no control group, which has been found to change without any intervention in previous de-implementation randomised controlled trials [18]. This makes conclusions on causality (i.e. P-KIP being responsible for the decrease in number of arthroscopies) debatable. We used interrupted time series analysis to mitigate this [28, 29, 31].

The basic patient demographics between those receiving arthroscopy and those in the P-KIP programme were different, with a tendency for more female and older patients in the P-KIP group. The reasons for this are not clear, however, it may represent a selection bias reflecting surgeon and/or patient perceived benefits of a lifestyle modification (for example, women with end stage knee osteoarthritis tend to have higher BMIs) [37].

We experienced a large number of patients not completing the face-to-face portion of the programme. This is, unfortunately, all too common within the modern NHS [38, 39]. We have implemented various strategies to mitigate this including giving patients a guide as the cost of the P-KIP [39]. It is possible this dropout rate leads to skewing of our patient outcome data, but not the arthroscopy data. Patient engagement in conservative care is likely to be an ongoing challenge.

Additionally, it is unclear if patients who drop out of the programme or complete it then go on to have an arthroscopy (i.e. if P-KIP delays rather than prevents arthroscopies). We have reported the number of arthroscopies for over 2 years from the date of P-KIP starting, with no apparent rebound to suggest this is the case. This would suggest these patients do not go on to have an arthroscopy in our institution (although it remains possible other institutions provide this operation).

The quality assurance aspect of our study demonstrates the improvement in patient outcome measures with intensive conservative care seen in this programme is consistent with other reports in the literature [5, 40]. However, it is unclear what form and how intensive conservative management must be (e.g. the number of sessions, telephone or face-to-face, group or individual) [41–43]. We also failed to capture change in weight of patients, despite having dietary and exercise interventions. Weight loss in key in managing knee OA [5], and BMI measures at follow up have been problematic to collect due to issues of practicality. However, the outcomes of this group of patients with conservative care is well reported and consistent across multiple studies, and therefore we feel confident that P-KIP is of a similar standard to conservative care pathways that reported elsewhere [2–7].

It is currently unclear what pathways patients take after completion of the programme (e.g. rate and timing of joint replacement). This work is being planned, but will take around 5–10 years to complete. What is known from the much publicised GRIFT report is that patients who have an arthroscopy for knee OA have a high rate of conversion to knee arthroplasty within a year [21]. Additionally, a proportion of patients, although improved after the programme, still demonstrated low scores. It may be that this represents a population that have failed conservative care, and require knee replacement. No set criteria are in place for such patients within P-KIP for referral on for knee replacement: the decision for this is made with the patient on a case-by-case basis. There is the potential for investigating factors that predict outcome, in the hopes of developing a model that could screen patients; however, we did not feel we had the necessary number of patients to produce precise estimates with regression modelling.

Finally, the original work on identifying barriers and designing the service was conducted in the same organisation that P-KIP has been piloted [15], and may mean the effect size seen in our organisation will be larger than that which will be achieved if the programme is taken up by other institutions. However, we feel that the barriers to implementation (e.g. unavailability of other options, time pressure, habit, perceived pressure to offer something) are common to most orthopaedic institutions. Nevertheless, some caution must be taken if transferring this programme to different institutions or to different healthcare systems. We feel very strongly that this programme has been a success due to the referral of a select group of patients, which in turn is due to engagement of orthopaedic surgeons. This engagement is key for expansion of the programme beyond our institution; we feel this programme is best used as an additional tool in the orthopaedic surgeons’ toolbox, allowing intensive conservative care for a select group of patients. Additionally, it is unclear how P-KIP would work in other healthcare systems, particularly systems where funding streams alter. Although there is no comparator group, we believe that the effect of P-KIP supports the premise that targeted interventions can deliver larger effects [16].

The economic effect of this programme is large with an estimated efficiency saving of approximately £300,000. This goes alongside decreasing pressure on operative capacity, which is commonly a cause of breaches in the 18-week target in NHS hospitals, a particular problem within orthopaedic NHS practice and one that can result in fines [21]. Such efficiency savings are part of the NHS budgetary plan.

Additionally, there are wider benefits as P-KIP generates health gains over and above improvement in knee function, as reflected in improvements in general health measures. This change likely reflects the multidisciplinary approach, including input from dietitians. Such health gains are likely to benefit patients over and above their knee related health.

## Conclusions

The result of this service evaluation suggests that the conservative care pathway P-KIP decreases the number of arthroscopies performed for knee osteoarthritis. Patients also experienced improvements in knee specific and general health outcomes. This project joins the body of evidence supporting the efficacy of interventions targeted to specific barriers, and the potential value of substitution strategies where alternative options are in short supply. It also provides an example of how interrupted time series analysis can be used in assessing the efficacy of interventions. With widespread dissemination, P-KIP has the potential to have an effect on efficiency savings, pressure on theatre capacity, and patient outcomes, although replication in other sites and the long-term effect is yet to be realised.

## Supplementary information

**Additional file 1.** Patient reported outcome measures A description of the patient reported outcome measures for the first cohort to complete P-KIP.

## Data Availability

Arthroscopy rates are presented in full in the results section, with outcome data supplied in the supplementary material. Breakdown of data is available on request to the corresponding author.

## Abbreviations

EQ 5D: Euroqol 5 Dimensions
OA: Osteoarthritis
OKS: Oxford Knee Score
P-KIP: Personalised Knee Improvement Programme
PSP: Perioperative Specialist Practitioner
