# Supplementary material for: Decreasing the number of arthroscopies in knee osteoarthritis – a service evaluation of a de-implementation strategy
Source: BMC Musculoskelet Disord. 2020 Mar 3;21:140. doi: 10.1186/s12891-020-3125-8 (PMC7055049; doi:10.1186/s12891-020-3125-8)
Supplement: Supplementary file 1 — Additional file 1. Patient reported outcome measures A description of the patient reported outcome measures for the first cohort to complete P-KIP. [file 12891_2020_3125_MOESM1_ESM.docx]

**Supplementary Material – Patient reported outcome measures**

This data is based on 377 patients who had completed the programme at the time of analysis, with outcome data available for 258 patients (68%). Forty-eight patients confirmed as self-discharging (13%), and the further 19% either patients either did not complete the face-to-face component of the study (ill health, movement out of area) or outcome data was not collected due to administration errors.

Functional and general health scores, collected at baseline and end of face-to-face contact was analysed using the Wilcox signed rank test, as scores were not normally distributed. Paired T-tests were also conducted, as t-tests are robust against deviations from normality,^28^ and provided identical results that are not displayed. Results are displayed in table 2.

|  | **Baseline scores median (IQR)** | **End of Face to face scores median (IQR); p-value** |
| --- | --- | --- |
| **OKS** | 26 (19-33) | 39 (31-43); p<0.001 |
| **EQ-5D Index score** | 70 (50-80) | 80 (70-90) ; p<0.001 |

*Outcome scores at baseline and end of face-to-face contact. IQR, inter quartile range; OKS, Oxford Knee score; EQ-5D, EuroQol 5-Dimensions.*
